# Minimum Clinically Important Difference (MCID) and Patient Acceptable Symptomatic State (PASS) Applied to the SF-36 in Patients Who Underwent Arthroscopic Rotator Cuff Repair

**DOI:** 10.3390/jcm13010178

**Published:** 2023-12-28

**Authors:** Umile Giuseppe Longo, Sergio De Salvatore, Ilaria Piergentili, Alberto Lalli, Benedetta Bandini, Vincenzo Denaro

**Affiliations:** 1 Fondazione Policlinico Universitario Campus Bio-Medico, Via Alvaro del Portillo, 200, 00128 Rome, Italy; s.desalvatore@unicampus.it (S.D.S.); albertolalli30@gmail.com (A.L.); benedettabandini.000@gmail.com (B.B.); denaro.cbm@gmail.com (V.D.); 2 Research Unit of Orthopaedic and Trauma Surgery, Department of Medicine and Surgery, Università Campus Bio-Medico di Roma, Via Alvaro del Portillo, 21, 00128 Rome, Italy; 3 Research Unit of Ospedale Pediatrico Bambin Gesù, Department of Medicine and Surgery, Via della Torre di Palidoro, 00050 Fiumicino, Italy; 4 CNR-IASI, Laboratorio di Biomatematica, Consiglio Nazionale delle Ricerche, Istituto di Analisi dei Sistemi ed Informatica, 00185 Rome, Italy; ilaria.piergentili94@gmail.com

**Keywords:** shoulder joint, rotator cuff repair, minimal clinically important difference, patient-reported outcome measures, patient acceptable symptomatic state

## Abstract

The 36-Item Short-Form Health Survey questionnaire (SF-36) is a reliable tool to assess the health-related quality of life of patients. If a mean difference between pre-operative evaluation and final follow-up is found to be statistically significant, then the change in score is not random. However, a statistically significant mean change may not correspond to a clinical amelioration for the patient or mean that the patient’s state of health is to be considered acceptable. For this reason, interest in the concepts of minimal clinically important difference (MCID) and patient acceptable symptomatic state (PASS) has grown within recent years. The goal of the present work of research was to determine the MCID and PASS values for the SF-36 in patients who received rotator cuff repair (RCR). Forty-six patients (18 women and 28 men, mean age 58.5 ± 12.9) previously diagnosed with rotator cuff disease were enrolled. All of these patients underwent RCR. They were evaluated pre-operatively and six months after the surgical intervention as a final follow-up. The SF-36 questionnaire was assessed at each evaluation. The MCID cut-offs of the total, physical, and mental dimensions of the SF-36 for patients who underwent RCR were 23.1, 32.5, and 18.1, respectively. A 23.1 improvement in the SF-36 score at six months following RCR can be correlated with patients having reached a clinically significant improvement in health status. If 81.9 or more is attained in the SF-36 score after surgical repair, the symptom state can be judged as satisfactory by the majority of patients.

## 1. Introduction

To assess rotator cuff repair (RCR), the most commonly applied clinical scores are the Disabilities of the Arm, Shoulder, and Hand Questionnaire, Shoulder Pain and Disability Index, American Shoulder and Elbow surgeons (ASES), Constant Murley Score, Simple Shoulder Test (SST), Oxford Shoulder Score, Shoulder Disability Questionnaire, and Western Ontario Shoulder Instability Index [1].

To determine the effectiveness of a given surgical procedure, the mean difference of an outcome score from baseline to postoperative follow-up is usually assessed. If the mean difference is statistically significant between the two time points, then the change in score is not random. However, a statistically significant mean variation may not correspond to a clinical improvement for the patient or mean that the patient’s state of health is to be considered acceptable. For this reason, interest in the concepts of Minimal Clinically Important Difference (MCID) and Patient Acceptable Symptomatic State (PASS) has increased over the last few years. The MCID is defined as the lowest amount of variation in an outcome measurement that enables a patient to self-assess their own level of improvement following therapy [2]. The PASS refers to the value that defines the patient’s satisfaction with a symptom state when the pain and function are considered together [3]. These tools can be exploited to evaluate if the outcomes considered in a research work or clinical environment for a surgical procedure can be judged as clinically meaningful.

The 36-Item Short-Form Health Survey questionnaire (SF-36) is a reliable tool to assess health-related quality of life, and it is broadly exploited in orthopedic-related research [4]. The SF-36 questionnaire is a 36-item health survey that considers eight different dimensions of health. These eight dimensions can be summarized into two synthetic indices: the first four compose the “physical component”, and the last four compose the “mental component” [5]. An additional one-item measure of self-evaluated change, from a year ago, in health status is also available. The raw scores are linearly translated into a scale with a minimum of 0 (worst condition) and a maximum of 100 (best condition), and the scores are evaluated using the Likert method for summed ratings. Also, the SF-36 dimensions are normalized into a scale ranging from 0 to 100.

Currently, to the author’s knowledge, no studies establishing the clinically relevant change and clinically acceptable symptom state in the SF-36 score in patients who underwent rotator cuff repair have yet been published. Therefore, the current study is the first work aiming to find the MCID and PASS values for the SF-36 score after RCR. It was hypothesized that an MCID and PASS threshold could be identified in patients undergoing RCR.

## 2. Materials and Methods

A total of 46 patients (18 males and 28 females) of mean age 58.5 ± 12.9 years, all previously diagnosed with rotator cuff tears of any grade, were included in the study. Prior to their inclusion, the entire cohort underwent a comprehensive assessment involving general medical history, physical examination, and radiological examinations of the shoulder. The repair procedures for these patients were performed between September 2019 and September 2020 at Campus Bio-Medico in Rome.

The severity of the tears was evaluated through preoperative magnetic resonance imaging and clinical examinations, which were conducted by two orthopedic surgeons. The lesions were graded according to the Goutallier [6] grade and Patte [7] classifications. Prior to undergoing arthroscopic repair, all patients had undergone conservative treatment, which encompassed physiotherapy and a rehabilitation regimen. All patients underwent surgical repair, consistently carried out by a single experienced surgeon, who performed arthroscopic rotator cuff repairs for all 46 patients. All tears were successfully repaired. Patients with shoulder conditions unrelated to those requiring surgical intervention were excluded from the study. Each patient adhered to a standardized rehabilitation protocol.

During the initial four weeks post-surgery, an abduction sling pillow was employed to support the arm, allowing only active elbow extension and flexion. By the fifth week, supervised physiotherapy sessions were initiated, occurring one to three times per week, supplemented by exercises to be performed independently during the remaining time. Between the fifth and eighth weeks, the rehabilitation process introduced passive forward elevation, passive external rotation, and active assisted range of motion (ROM) exercises, gradually progressing to active ROM exercises. From the eighth to the tenth week, the rehabilitation regimen shifted towards strengthening the deltoid and scapular stabilizers, alongside concentric and eccentric exercise protocols targeting the rotator cuff muscles. The assessment of patients included administration of the SF-36 questionnaire during each evaluation time point.

The study was conducted according to the guidelines of the Declaration of Helsinki and was approved by the Institutional Review Board of Campus Bio-Medico University of Rome (COSMO study, Protocol number: 78/18 OSS ComEt CBM, 16/10/18).

### 2.1. Assessment Instruments

Six months after the rotator cuff repair, patients were presented with the following queries related to their satisfaction: “How do you feel after the surgery you have undergone?” and “Compared to a year ago, how do you feel?” to determine the MCID [8]. Two anchors were used to verify the consistency of results. For both questions, the available answers were “much worse”, “a little worse”, “equal”, “a little better” and “much better”. Patients who replied with “much worse”, “a little worse” or “equal” were grouped into the “no-change” cohort, while patients who replied with “a little better” represented the “minimally improved” group. To calculate the PASS, patients were asked the question “Does shoulder pain affect the performance of your usual work activities (including domestic ones)?” at final evaluation. The potential answers were “Yes” or “No”. An acceptable symptom state was attributed to patients who answered “No”.

### 2.2. Statistical Analysis

A priori power analysis with an effect size of 0.71 (Cohen’s d) [9], an alpha level of 0.05 (two-tailed), and a power of 0.80 revealed a minimum sample of 18 patients. Data normality for the SF-36 score and physical and mental dimensions of SF-36 at baseline and last follow-up using the Shapiro–Wilk test was established. Values at initial and final evaluations were compared using the Wilcoxon Signed Rank Test. The threshold for statistical significance was established at 0.05. SPSS version 26 for Windows (SPSS Inc., Chicago, IL, USA) was exploited for all statistical analyses.

The MCIDs for the SF-36 were estimated using a variety of anchor approaches and distribution-based methods. The following distribution-based tools were applied: 0.5 Standard Deviation (0.5 SD), the Standard Error of Measurement (SEM), and the Minimum Detectable Change (MDC). The 0.5 SD is correlated to effect size (0.5 SD is a median effect). The MDC correlates with the smallest variation above the measurement error with a confidence interval, while the SEM is the smallest variation above the measurement error (usually 95% confidence). For the present statistical analysis, Cronbach’s alpha was applied as the measure of the reliability of the SF-36 in determining the SEM and MDC. The surgical satisfaction questionnaire was administered to patients for use as an anchor. Receiver Operating Characteristic (ROC) curves were exploited to determine the variation in SF-36 cut-off with maximized sensitivity and specificity. The Area Under the Curve (AUC) is considered the standard measure to assess the accuracy. It ranges in value from 0.5 (no better than chance) to 1.0 (perfect discrimination). Values of AUC less than 0.5 were considered not valid, and values of AUC less than 0.7 were considered not acceptable [10]. The MCID was also assessed via the Mean Change in SF-36 from initial evaluation (prior to intervention) to follow-up after the surgical procedure in patients who reported an improvement in surgical satisfaction. The pain questionnaire was used to calculate the PASS of SF-36. The 75th percentile of patients’ cumulative percentage curve who self-assessed their symptom state to be satisfying was used to determine the PASS values of the SF-36 (patients who responded “No” at the pain questionnaire) and the point on the ROC curve, for which the threshold was calculated via the Youden index.

## 3. Results

Non-normal distribution of the SF-36 at initial screening and final evaluation was assessed using the Shapiro–Wilk test (*p* < 0.05).

At the initial evaluation, the average SF-36 score was 52.7 ± 17.2 (range 17.8–85.5) (0% floor and ceiling effects). At six months, the mean SF-36 score was 81.8 ± 14.6, ranging from 25.1 to 95.1 (0% floor and ceiling effects). A statistically relevant mean variation between initial and final evaluations was determined (*p* < 0.001). At baseline, the average physical component of the SF-36 score was 53.5 ± 17.8, ranging from 22.8 to 84.4 (0% floor and ceiling effects). At final follow-up, the mean physical component of the SF-36 score was 83.6 ± 17.6, ranging from 27.9 to 98.8 (0% floor and ceiling effects). A statistically significant mean variation between initial and final evaluations was found (*p* < 0.001).

At baseline, the average mental component of the SF-36 score was 65.6 ± 22.2 (range 16.9–95.5) (0% floor and ceiling effects). Six months post-operatively, the mean mental component of the SF-36 score was 84.1 ± 15, ranging from 28.6 to 98.8 (0% floor and ceiling effects). A statistically significant mean variation between initial and final screenings was assessed (*p* < 0.001).

The internal consistency reliability for the total, physical, and mental dimensions of the SF-36 was high (α = 0.9) (Table 1).

### 3.1. Total, Physical, and Mental Components of SF-36

MCID estimates for the SF-36 for patients who underwent RCR ranged from 3.5 to 23.1. The following MCIDs with distribution-based approaches were computed: an MCID of 7.6 (0.5 SD) with a medium effect size (ES = 0.5); an MCID of 3.5 (SEM) with an internal consistency reliability of 0.9; an MCID of 9.6 (MDC) at the 95% confidence level (Table 2).

### 3.2. MCID of Physical Dimension of SF-36

MCID estimates for the physical dimension of the SF-36 for patients who underwent RCR ranged from 5.6 to 32.5. The following MCIDs with distribution-based approaches were computed: an MCID of 9.6 (0.5 SD) with a medium effect size (ES = 0.5); an MCID of 5.6 (SEM) with an internal consistency reliability of 0.9; an MCID of 15.4 (MDC) at the 95% confidence level (Table 2).

### 3.3. MCID of Mental Dimension of SF-36

MCID estimates for the physical dimension of the SF-36 for patients who underwent RCR ranged from 5.9 to 18.1. The following MCIDs with distribution-based approaches were computed: an MCID of 9.1 (0.5 SD) with a medium effect size (ES = 0.5); an MCID of 5.9 (SEM) with an internal consistency reliability of 0.9; an MCID of 16.4 (MDC) at the 95% confidence level (Table 2).

### 3.4. PASS Values of SF-36

The SF-36 score with a PASS value of 81.9 had the highest levels of sensitivity and specificity. With the 75th percentile approach, 90.6 was the threshold. The PASS values of the physical dimension of the SF-36 were 88.3 and 94.5, using ROC and 75th percentile tools, respectively. The PASS values of the mental component of the SF-36 were 82 and 94.4, using ROC and 75th percentile approaches, respectively. All of these ROC calculations show high instrument responsiveness (AUC = 0.9, AUC = 0.9, and AUC = 0.8) (Table 3).

## 4. Discussion

The main finding of this article is that an improvement of 23.1 in the SF-36 score after a rotator cuff repair shows that patients’ health improved in a way that is clinically significant. If 81.9 or more is attained in the SF-36 score after surgical repair, the majority of patients will find the symptom state satisfying.

A consistent technique for assessing patients’ mental and physical health was created with the SF-36 survey. The clinical responsiveness of this questionnaire has been previously assessed by several authors [11,12,13,14]. However, MCIDs or PASS estimations for individuals with shoulder pathology have only been reported by a small number of authors. MCIDs and PASS have been reported in relation to several clinical scores, other than the SF-36, for patients who underwent RCR [15,16,17,18,19,20,21]. Among those, Cvetanovich and colleagues [18] provided essential insights into the MCID, substantial clinical benefit (SCB), and PASS thresholds in the context of individuals undergoing arthroscopic rotator cuff repair. Specifically, their study indicated that the corresponding threshold values for the ASES score were 11.1 for MCID, 17.5 for SCB, and 86.7 for PASS. Also, Tashjian et al. stated that a change at least of 4.3 in the SST score from pre-operative to post-operative time points corresponds to a minimal clinical improved in the patient [22]. At the same time, there have been several publications determining the MCID values in different surgical settings, such as total knee replacement, total hip replacement, and hip arthroscopic surgery [23,24,25,26,27,28,29]; however, no studies have yet evaluated the MCID and PASS of the SF-36 for patients undergoing RCR.

In the current literature, MCID estimates ranged between 3.3 and 24.9 for the SF-36. In the present study, the MCID for the SF-36 ranged from 3.5 to 23.1. It has been reported that a legitimate MCID should be at least bigger than the SEM value and depict how significant the patient believes the improvement (or worsening) to be [30]. Furthermore, as reported by Stipancic et al. [31], a useful MCID should be greater than the MDC. Therefore, the MCIDs calculated with the ROC method were useless because they were less than the MDC values [32,33,34]. Furthermore, since the AUC of the mental dimension of the SF-36 was less than 0.7, the MC method seems to be the most reliable in quantifying MCID values.

The MCID cut-offs of the total, physical, and mental dimensions of the SF-36 for patients who underwent RCR were 23.1, 32.5, and 18.1. These findings suggest that a variation greater than 9.6 for the SF-36, 15.4 for the physical dimension of the SF-36, and 16.4 for the mental dimension of the SF-36 (the MDC value) demonstrates that the change is not likely the result of random variation, while a change larger than 23.1 for the SF-36, 32.5 for the physical dimension of the SF-36, and 18.1 for the mental dimension of the SF-36 (the MCID values) reflects a clinically relevant variation.

The PASS for the SF-36 has not previously been estimated in any orthopedic study. This is the first paper in which the PASS for the SF-36 was calculated for patients who underwent RCR. The total SF-36 cut-offs were 81.9 (AUC = 0.9) with the ROC tool and 90.6 with the 75th percentile approach. The PASS values of SF-36′s physical dimension were 88.3 (AUC = 0.9) and 94.5, while for the mental dimension they were 82 (AUC = 0.8) and 94.4, using the ROC and 75th percentile methods, respectively. Given the high value of AUC, the ROC tool appears to be the most accurate. Therefore, the PASS values of the total, physical, and mental dimensions of the SF-36 for patients who underwent RCR were 81.9, 88.3, and 82.

This study relies on several points of strength. This the first article that calculated the MCID and the PASS values of the SF-36 in patients who underwent RCR. Secondly, the MCID and PASS cut-offs were only determined using ad hoc techniques. Furthermore, both distribution and anchor approaches were exploited to establish the MCID, and to verify the consistency of results, two anchors were used to quantify the MCID.

Nonetheless, the current work also presents some weaknesses. The MCID and PASS were computed from baseline to a post-operative evaluation occurring only six months after the intervention and therefore cannot deliver long-term objective data. We have partially addressed this point of vulnerability by taking into consideration the long-term findings. These findings provide minimal evidence of alteration in SF-36 scores beyond the initial six-month period subsequent to RCR, extending up to a duration of 2 years. It is possible that the MCID thresholds may vary according to the different timing of the follow-up evaluation. The power analysis ascertained the adequacy of the enrolled patient count; nonetheless, it was grounded in a more substantial effect size than what was observed. Within this domain, it is customary to employ larger participant cohorts in published studies, and the limited patient population under evaluation in this review introduces the possibility of inherent bias.

## 5. Conclusions

The hypothesis of the current study was that MCID and PASS for patients undergoing RCR could be identified. A 23.1 improvement in the SF-36 score after RCR indicates that patients have achieved a clinically relevant amelioration in their health status. If 81.9 or more is attained in the SF-36 score after surgical repair, the majority of patients will consider their symptom state satisfactory.

## Figures and Tables

**Table 1:** Summary of total, physical, and mental dimensions of the SF-36 score at baseline and six months’ follow-up.

Score	Baseline Follow-Up			6-Month Follow-Up		
	Mean ± SD	Minimum	Maximum	Mean ± SD	Minimum	Maximum
Total SF-36	52.7 ± 17.2	17.8	85.5	81.8 ± 14.6	25.1	95.1
Physical SF-36	53.5 ± 17.8	22.8	84.4	83.6 ± 17.6	27.9	98.8
Mental SF-36	65.6 ± 22.2	16.9	95.5	84.1 ± 15	28.6	98.8

SF-36, 36-item short-form health survey questionnaire; SD, standard deviation.

**Table 2:** MCID of SF-36 and dimensions of SF-36 in patients who underwent rotator cuff repair.

Score	0.5 SD	SEM	MDC	ROC (AUC)	Mean Change
Total SF-36	7.6	3.5	9.6	8.2 (0.8)	23.1
Physical SF-36	9.6	5.6	15.4	4.8 (0.9)	32.5
Mental SF-36	9.1	5.9	16.4	12 (0.6)	18.1

SF-36, 36-item short-form health survey questionnaire; SD, standard deviation; SEM, standard error of measurement; MDC, minimum detectable change; ROC, receiver operating characteristic; AUC, area under the curve.

**Table 3:** PASS of SF-36 and dimensions of SF-36 in patients who underwent rotator cuff repair.

Score	ROC (AUC)	75th Percentile
Total SF-36	81.9 (0.9)	90.6
Physical SF-36	88.3 (0.9)	94.5
Mental SF-36	82 (0.8)	94.4

SF-36, 36-item short-form health survey questionnaire; ROC, receiver operating characteristic; AUC, area under the curve.

## Data Availability

The data presented in this study are available on request from the corresponding author.
